# Mechanisms underlying the therapeutic effects of cinobufagin in treating melanoma based on network pharmacology, single-cell RNA sequencing data, molecular docking, and molecular dynamics simulation

**DOI:** 10.3389/fphar.2023.1315965

**Published:** 2024-01-29

**Authors:** Jiansheng Yang, Chunchao Cheng, Zhuolin Wu

**Affiliations:** ^1^ Department of Dermatology, The Peoples Hospital of Yudu County, Ganzhou, China; ^2^ Department of Neurosurgery, Tianjin Medical University General Hospital, Laboratory of Neuro-oncology, Tianjin Neurological Institute, Key Laboratory of Post-Neuro Injury Neuro-Repair and Regeneration in Central Nervous System, Ministry of Education, and Tianjin City, Tianjin, China

**Keywords:** melanoma, cinobufagin, network pharmacology, EGFR, ERBB2, CDK2

## Abstract

Malignant melanoma is one of the most aggressive of cancers; if not treated early, it can metastasize rapidly. Therefore, drug therapy plays an important role in the treatment of melanoma. Cinobufagin, an active ingredient derived from Venenum bufonis, can inhibit the growth and development of melanoma. However, the mechanism underlying its therapeutic effects is unclear. The purpose of this study was to predict the potential targets of cinobufagin in melanoma. We gathered known and predicted targets for cinobufagin from four online databases. Gene Ontology (GO) analysis and Kyoto Encyclopedia of Genes and Genomes (KEGG) enrichment analysis were then performed. Gene expression data were downloaded from the GSE46517 dataset, and differential gene expression analysis and weighted gene correlation network analysis were performed to identify melanoma-related genes. Using input melanoma-related genes and drug targets in the STRING online database and applying molecular complex detection (MCODE) analysis, we identified key targets that may be the potential targets of cinobufagin in melanoma. Moreover, we assessed the distribution of the pharmacological targets of cinobufagin in melanoma key clusters using single-cell data from the GSE215120 dataset obtained from the Gene Expression Omnibus database. The crucial targets of cinobufagin in melanoma were identified from the intersection of key clusters with melanoma-related genes and drug targets. Receiver operating characteristic curve (ROC) analysis, survival analysis, molecular docking, and molecular dynamics simulation were performed to gain further insights. Our findings suggest that cinobufagin may affect melanoma by arresting the cell cycle by inhibiting three protein tyrosine/serine kinases (EGFR, ERBB2, and CDK2). However, our conclusions are not supported by relevant experimental data and require further study.

## 1 Introduction

Malignant melanoma is the most aggressive form of skin tumor, developing from melanin-producing melanocytes (Leonardi et al., 2018). It can develop from multiple nevi and, once formed, invasion and metastasis can occur rapidly (Spagnolo et al., 2019). Genetic mutation is one of the primary drivers in the occurrence and development of melanoma, including oncogene mutations (RAS, BRAF, ALK, and MET) and tumor suppressor mutations (TP53 and CDKN2A) (Tsao et al., 2012). Moreover, several signaling pathways are also implicated in the growth and progression of malignant melanoma, such as PI3K-AKT, RAS-RAF-MEK-ERK, and the canonical Wnt/β-catenin signaling pathway (Lopez-Bergami et al., 2008). Therefore, multiple mechanisms are involved in the development of melanoma. After diagnosis, the main treatment method for early non-metastatic melanoma is surgical resection. However, in the case of advanced malignant melanoma, which often has metastases, a comprehensive and multidisciplinary approach should be applied, such as chemotherapy, radiation, and immunotherapy (Singh et al., 2017). Therefore, it is necessary to find new targets and novel therapeutics for melanoma treatment.

Traditional Chinese medicine (TCM) is a valuable body of knowledge that has made significant contributions to human health worldwide (Dashtdar et al., 2016). Cinobufagin is one of the active components of Venenum Bufonis, a traditional Chinese medicine (Chen et al., 2013). Although cinobufagin was originally used as a painkiller to relieve cancer pain, it can also inhibit the growth of many kinds of tumors. The Chinese State Food and Drug Administration has approved cinobufagin for the treatment of liver and prostate cancer (Meng et al., 2009). Cinobufagin can apparently induce apoptosis and cell cycle arrest in several tumor types, including melanoma (Cui et al., 2010; Qi et al., 2011; Chen et al., 2013; Lu et al., 2016; Zhu et al., 2017; Pan et al., 2019; Zhao et al., 2019). Nonetheless, the underlying mechanism of cinobufagin’s effects in malignant melanoma is not well understood.

Network pharmacology is a useful strategy for exploring the underlying mechanisms related to cancer development and the effects of drugs. In our study, we aim to investigate the mechanism of the effect of cinobufagin in melanoma treatment by employing network pharmacology, transcriptional sequencing data analyses, molecular docking, and molecular dynamics simulation.

## 2 Materials and methods

### 2.1 Identification of targets of cinobufagin

The SwissTargetPrediction online database (https://www.swisstargetprediction.ch/) and ChEMBL online database (https://www.ebi.ac.uk/chembldb/) were used to inquire about cinobufagin’s known and possible targets. The Comparative Toxicogenomics Database (CTD; https://ctdbase.org/) and SuperPred database (http://prediction.charite.de/) were also used to predict possible targets of cinobufagin. The functional enrichment analyses of these drug targets were displayed by the “clusterProfiler” package of the R Programming Language, including Gene Ontology (GO) and Kyoto Encyclopedia of Genes and Genomes (KEGG) (Yu et al., 2012). The three categories of GO analysis for these drug targets were identified, namely, cellular component (CC), biological process (BP), and molecular function (MF), to examine the biological characteristics of these drug targets. KEGG enrichment was used to identify potential signaling pathways.

### 2.2 Identification of DEGs in melanoma

A total of 83 disease samples and 17 healthy samples in the GSE46517 microarray dataset were acquired from the GEO online database (http://www.ncbi.nlm.nih.gov/geo). First, row data were normalized. Using R software’s “limma” package (Ritchie et al., 2015), differentially expressed genes (DEGs) between disease samples and healthy controls were identified with the following criterion: *p* < 0.05 and |fold change|(FC) > 1. Volcano and heatmap plots were generated to show the DEGs and the significant genes were labeled. Gene set enrichment analysis (GSEA) was performed to identify defined genomes (Subramanian et al., 2007). Through GSEA analysis, the differences between the two biological processes of DEGs were identified.

### 2.3 Weighted gene co-expression network analysis

The WGCNA package of R was used to construct a gene co-expression network in GSE46517 (Langfelder and Horvath, 2008). A hierarchical clustering tree was generated to dispose of the outlier sample. Then, the topological overlap and correlation matrices between genes were calculated. The “pickSoftThreshold” function of the WGCNA package was used to compute the soft threshold power. Through a set screening threshold, we converted the paired correlation matrix into a neighborhood correlation matrix to ensure that the scale-free network individually calculated the paired Pearson correlation coefficients between all genes. The eigenvector values were calculated for each module. We then converted the adjacency matrix to a topological overlap matrix (TOM), computed the corresponding dissimilarity, and conducted a hierarchical clustering analysis. Lastly, we measured the connection between the gene modules and people, normal and abnormal, via gene significance (GS) values and module membership (MM) values and then identified the key modules.

### 2.4 Generation of protein–protein interaction networks and identification of key clusters

Using the STRING website (https://string-db.org/), the protein–protein interactions (PPIs) of drug targets and melanoma-related genes were investigated. The network nodes and edges of PPIs performed interactions among these proteins. We used Cytoscape software to further optimize the PPI networks, and the molecular complex detection (MCODE) algorithm was performed to screen the key targets that contribute to melanoma growth and proliferation.

### 2.5 Differential expression of key targets in melanoma and normal tissues

By putting key targets back into the GSE46517 dataset to identify the differential expression of the key cluster between melanoma and control groups, the screening conditions were *p*-value < 0.05 and |[(log2 fold-change)]| > 1. TCGA melanoma data were downloaded from the UCSC XENA dataset (https://xena.ucsc.edu/). Differential expression of the key targets in TCGA melanoma data was also conducted.

### 2.6 Single-cell RNA sequencing data analysis and identification of melanoma-associated genes

The row dates of GSE215120 performed in the analysis were downloaded from the GEO online database. We chose six acral melanoma samples for our analysis. Using the Seurat package of R software, we processed data with strict criteria: min.cells = 3, min.features = 200, af$nFeature_RNA ≥ 200 and af$nFeature_RNA ≤ 5,000, af$percent.mt ≤ 20, and af$percent.rb ≤ 20. After cells were filtered based on the above criterion, cells for data analysis were clustered and visually classified using the unified manifold approximation and projection dimensionality reduction techniques. We used R to show the distribution in the “singscore” of pharmacological key targets on the cell subtype.

### 2.7 Receiver operating characteristic curve analysis and survival analysis

RNA sequencing and survival data on melanoma patients were downloaded from the TCGA public database (http://xena.ucsc.edu/). The crucial targets were identified at the intersection of key clusters, melanoma-related targets, and drug-related genes. Receiver operating characteristic (ROC) curves of these crucial targets were plotted, and these targets were evaluated by computing the area under the ROC curve. We selected the overall survival time of the patients to construct the Kaplan–Meier survival curve and used all three tests (Log–rank, Breslow, and Tarone–Ware) to compare the significant differences between the curves in the graph. Overall survival time is the time from the start of treatment for melanoma patients to the time of death. The censored cases were displayed as “+” on the survival curve.

### 2.8 Molecular docking and molecular dynamics simulation

The crystal structures of EGFR, ERBB2, and CDK2 were downloaded from the PDB website (EGFR:5FED, ERBB2:3PP0, and CDK2:1B39). For better simulation, protein structures containing active site inhibitors were preferentially selected. The structure of cinobufagin was obtained from ChemDraw. First, the protein was executed using “add hydrogen” and “clean up” in Discovery Studio 2019, and the ligand was also prepared with this tool. We utilized the primitive ligand to provide the binding site. The CDOCKER function was then used to perform molecular docking and calculate -cdocker interaction energy. CDOCKER is a docking method with rigid protein and flexible ligand; -cdocker interaction energy can reflect the energy of the ligand–protein interaction—the higher the score, the stronger the bond. We used Discovery Studio to calculate the binding energy. Molecular dynamics simulation was performed with the Gromacs2020 package. The CHARMm36 force field was employed to execute a molecular dynamics simulation. The system was dissolved in TIP3P water molecules in a dodecahedral box. Energy minimization and NVT and NPT simulations were then performed on the system. Finally, a 50-ns-long molecular dynamics simulation was performed, and root mean square deviation (RMSD) values were calculated.

### 2.9 Statistical analysis

All statistical analyses in the present research were implemented using R software (version 4.3.1). *p* < 0.05 was used as the threshold for statistical significance.

## 3 Results

### 3.1 General targets of cinobufagin

**FIGURE 1:**
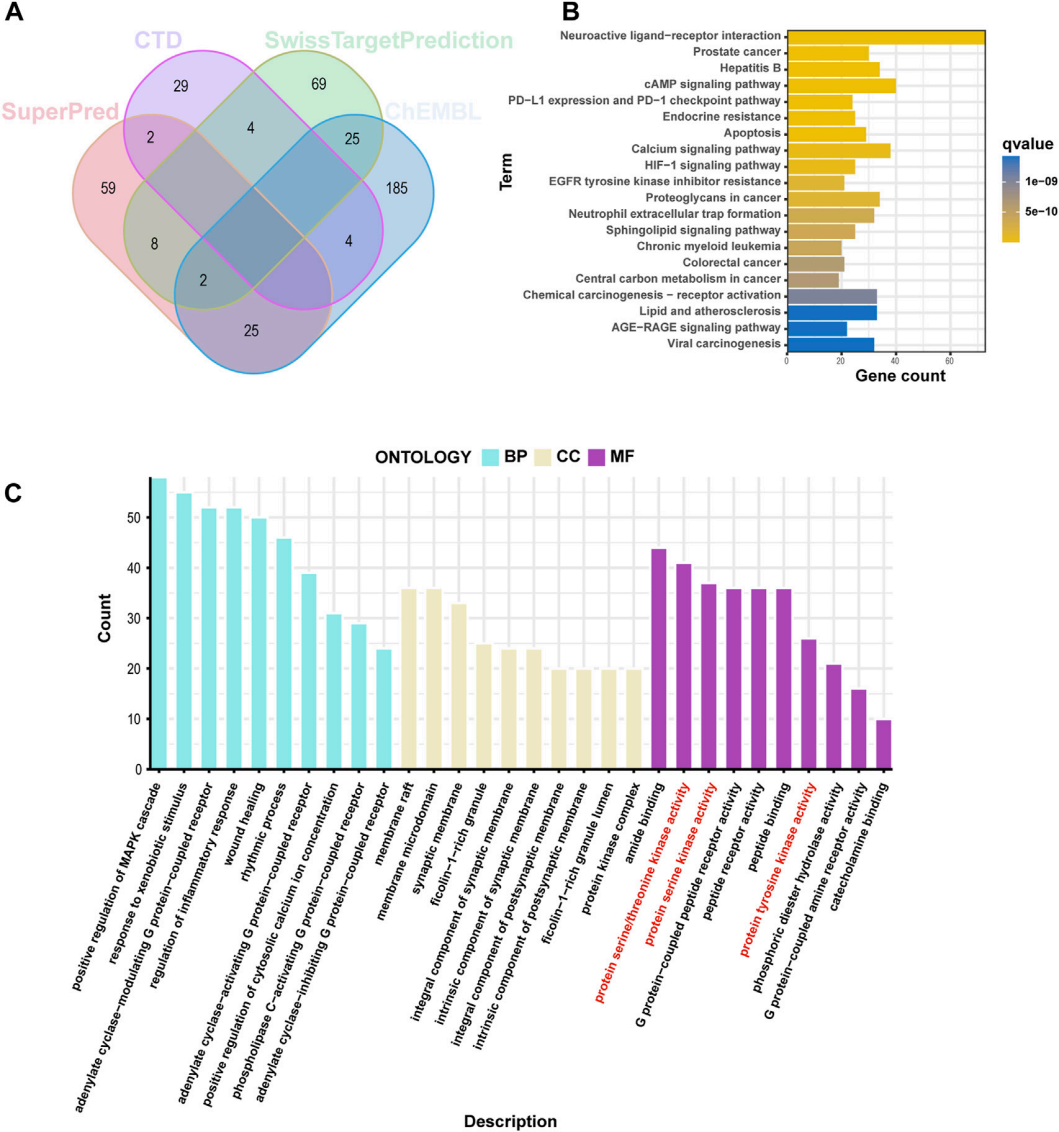
Screening analysis of cinobufagin targets. **(A)** Venn diagram of cinobufagin in the four databases (CTD: Comparative Toxicogenomics database). **(B)** Kyoto Encyclopedia of Genes and Genomes (KEGG) enrichment analysis of cinobufagin targets. **(C)** Gene Ontology (GO) enrichment analysis (BP, biological process; CC, cellular component; MF, molecular function) of cinobufagin targets.

We identified 108 and 241 related targets of cinobufagin from the SwissTargetPrediction and ChEMBL databases, respectively. Through CTD and the SuperPred database, we predicted 39 and 96 additional potential drug targets, respectively (Figure 1A). These 413 drug-related targets were used for GO and KEGG analyses. The biological process category in GO was mainly enriched in positive regulation of the MAPK cascade, response to xenobiotic stimulus, and the adenylate cyclase-modulating G protein-coupled receptor signaling pathway. The GO cellular component category was mainly enriched in the membrane raft, membrane microdomain, and synaptic membrane. The molecular function section was mainly gathered in amide binding, protein serine/threonine kinase activity, and protein serine kinase activity (Figure 1C). KEGG pathway enrichment of these drug targets was mainly gathered in neuroactive ligand–receptor interaction, prostate cancer, hepatitis B, and the cAMP signaling pathway in cancer (Figure 1B). In total, these results suggest that cinobufagin might be regulating protein serine/threonine kinase activity.

### 3.2 Target genes in melanoma

**FIGURE 2:**
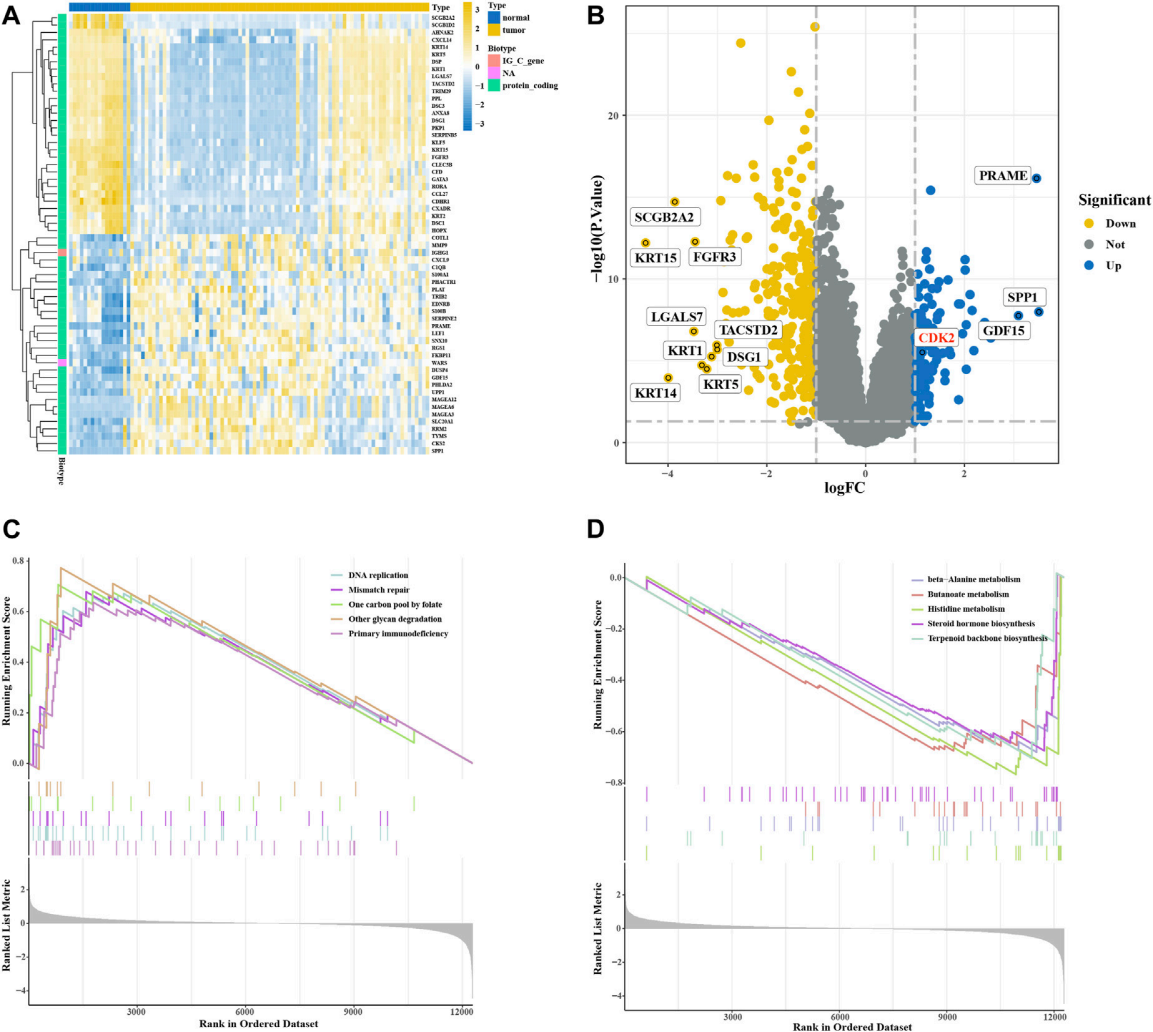
Expression of differentially expressed genes (DEGs) in the GSE46517 dataset. **(A)** Heatmap plot of DEGs in the GSE46517 dataset. **(B)** Volcano plot of DEGs in the GSE4290 dataset. **(C,D)** GSEA analysis based on KEGG analysis.

We downloaded 100 melanoma-related samples from GSE46517 from the GEO database, including 8 normal skin tissues, 9 nevus tissues, 31 primary melanoma tissues, and 52 metastatic melanoma tissues. We normalized raw sequencing data first and then identified DEGs between control groups (including normal skin tissues and nevus tissues) and melanoma groups (including primary melanoma tissues and metastatic melanoma tissues) (Supplementary Figure S1). Among these genes, 105 were upregulated and 285 were downregulated (Supplementary Table S1). The heatmap plot showed 60 significant DEGs, and the top 12 DEGs were shown in the volcano plot (Figures 2A, B). GSEA analysis was used to evaluate the pathway enrichment of DEGs between the melanoma and normal groups. The results of this analysis showed that DNA replication, mismatch repair, one-carbon pool by folate, other glycan degradation, and primary immunodeficiency were enriched in melanoma groups (Figure 2C). The beta-alanine metabolism, butanoate metabolism, histidine metabolism, steroid hormone biosynthesis, and terpenoid backbone biosynthesis were inhibited (Figure 2D). These results suggest that DNA replication may play an important role in melanoma development.

### 3.3 WGCNA analysis

**FIGURE 3:**
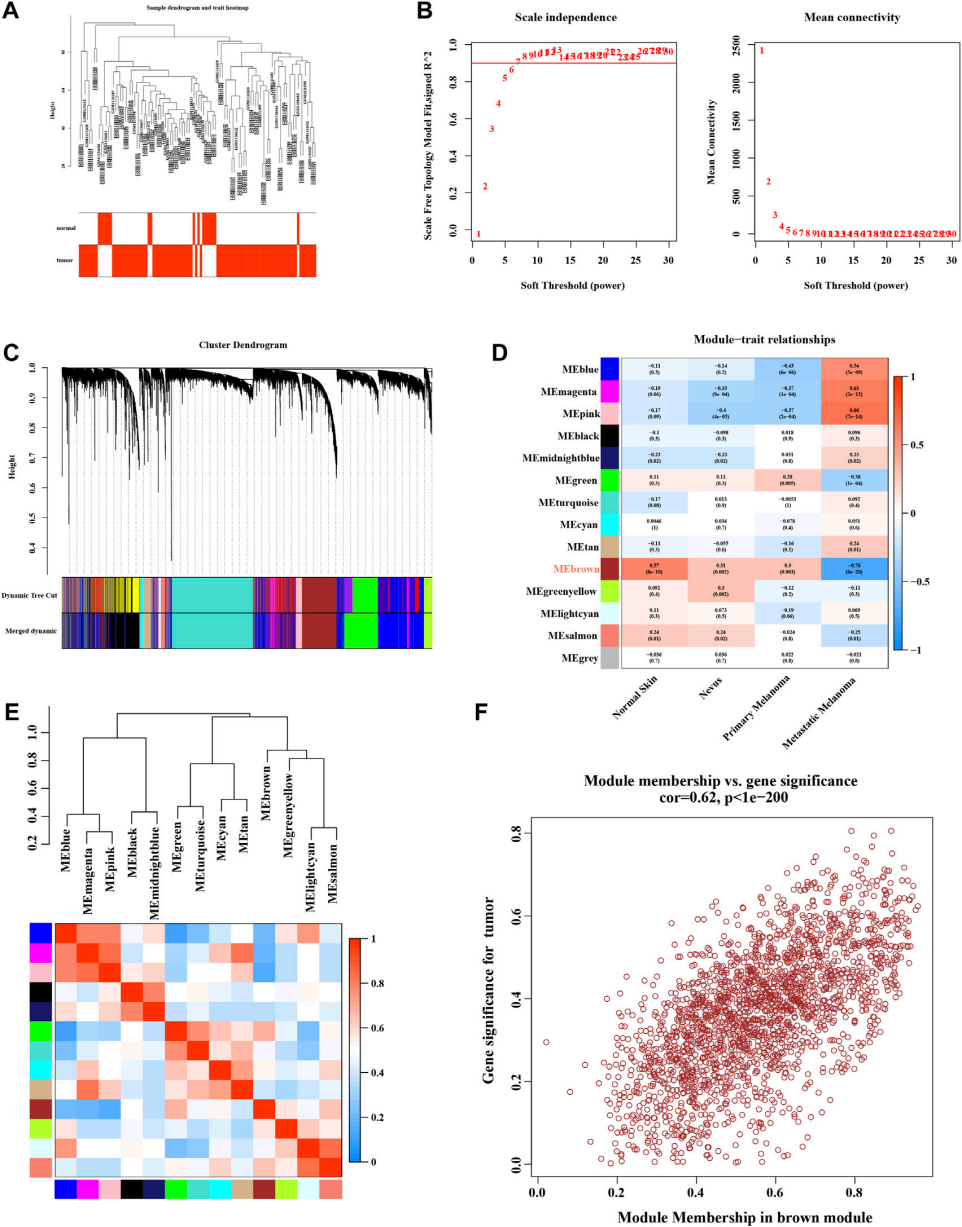
Enrichment levels in genomic weighted gene co-expression network analysis (WGCNA). **(A)** Sample dendrogram and trait heatmap. **(B)** Selection of soft thresholds. **(C)** Cluster dendrogram of WGCNA. **(D)** Correlations between gene modules and melanoma status. **(E)** Correlation between modules. **(F)** Correlation between brown module memberships and gene significance.

We used microarray data on GSE46517 for WGCNA analysis. The outlier detection indicated no significant outliers in the data (Figure 3A). The soft threshold power was evaluated as 6 with a scale-free index of 0.9, indicating that connectivity was reasonable (Figure 3B). The topological overlap matrix and correlation matrix between the data genes were constructed. The co-expression network was then established, and the cluster dendrogram with a dynamic tree cut and merged dynamic plot was constructed (Figure 3C). Finally, the results of data clustering were divided into 14 modules (Figure 3D). The correlation coefficient between each module and melanoma-related phenotype was calculated. The results suggested that the MEbrown module was the one most significantly related to primary (cor = 0.3, *p* = 0.003) and metastatic (cor = −0.76, *p* = 6e-20) melanoma. The correlation heatmap between these modules is shown in Figure 3E. The scatter plot of module membership (MM) and gene significance (GS) showed excellent correlation within the MEbrown module (R = 0.62, *p* < 1e-200) (Figure 3F). Therefore, the MEbrown module could be an optimized module to explain the anomalous melanoma phenotypes.

### 3.4 Identification of key targets

**FIGURE 4:**
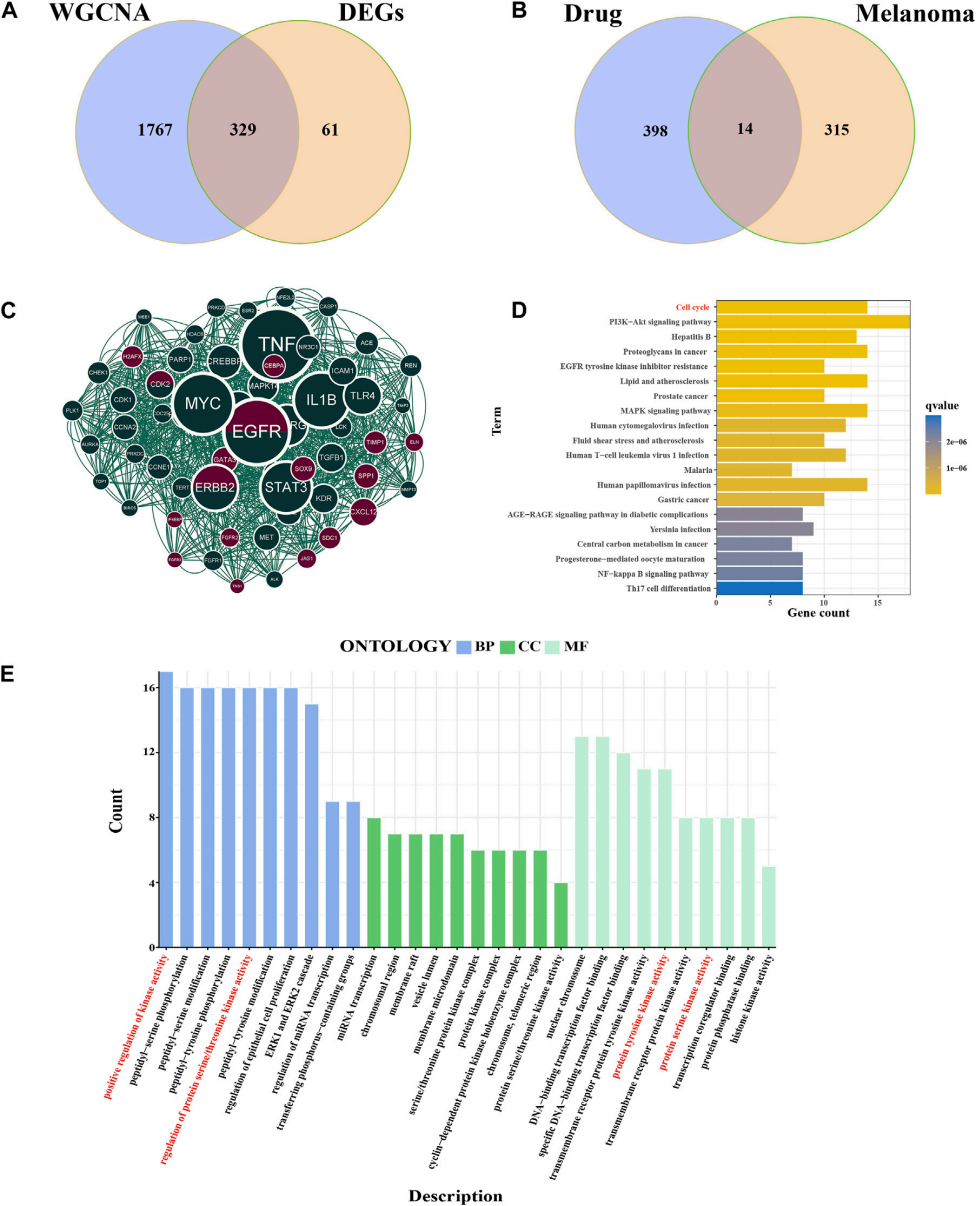
Identification of key targets and functional analysis. **(A)** Intersection of DEGs and WGCNA brown module genes, named melanoma-related genes. **(B)** Intersection of melanoma-related genes and drug targets. **(C)** Cytoscape’s plugin code for all melanoma-related genes and drug targets, named key cluster. Deep green: melanoma-related gene; brown: drug targets; deep green and brown: both melanoma-related genes and drug targets. **(D)** KEGG analysis of key genes. **(E)** GO analysis of the key cluster.

We compared the DEGs and MEbrown module genes to identify 329 melanoma-related genes (Figure 4A). 14 genes were identified in intersection between melanoma-related genes and drug targets (Figure 4B). The PPI network of all melanoma-related and drug-related genes was constructed using the STRING online database (Supplementary Figure S2). The molecular complex detection (MCODE) algorithm was used to identify 62 essential subpopulation genes, termed key targets or key clusters (Figure 4C). The GO analysis of key targets showed that the biological process category was mainly enriched in the positive regulation of kinase activity, peptidyl-serine phosphorylation, and peptidyl-serine modification. The Biological Process category shown that the key cluster mainly enriched in miRNA transcription, and chromosomal region, and membrane raft. The results of the molecular function section were gathered in nuclear chromosome, DNA-binding transcription factor binding, and specific DNA-binding transcription factor binding (Figure 4E). Through KEGG-enriched analysis, we found that these key targets were mainly gathered in the cell cycle, PI3K-Akt signaling pathway, and hepatitis B (Figure 4D). Notably, the cell cycle was one of the primary signaling pathways affected by cinobufagin in cancer.

### 3.5 The expression and distribution of key targets

**FIGURE 5:**
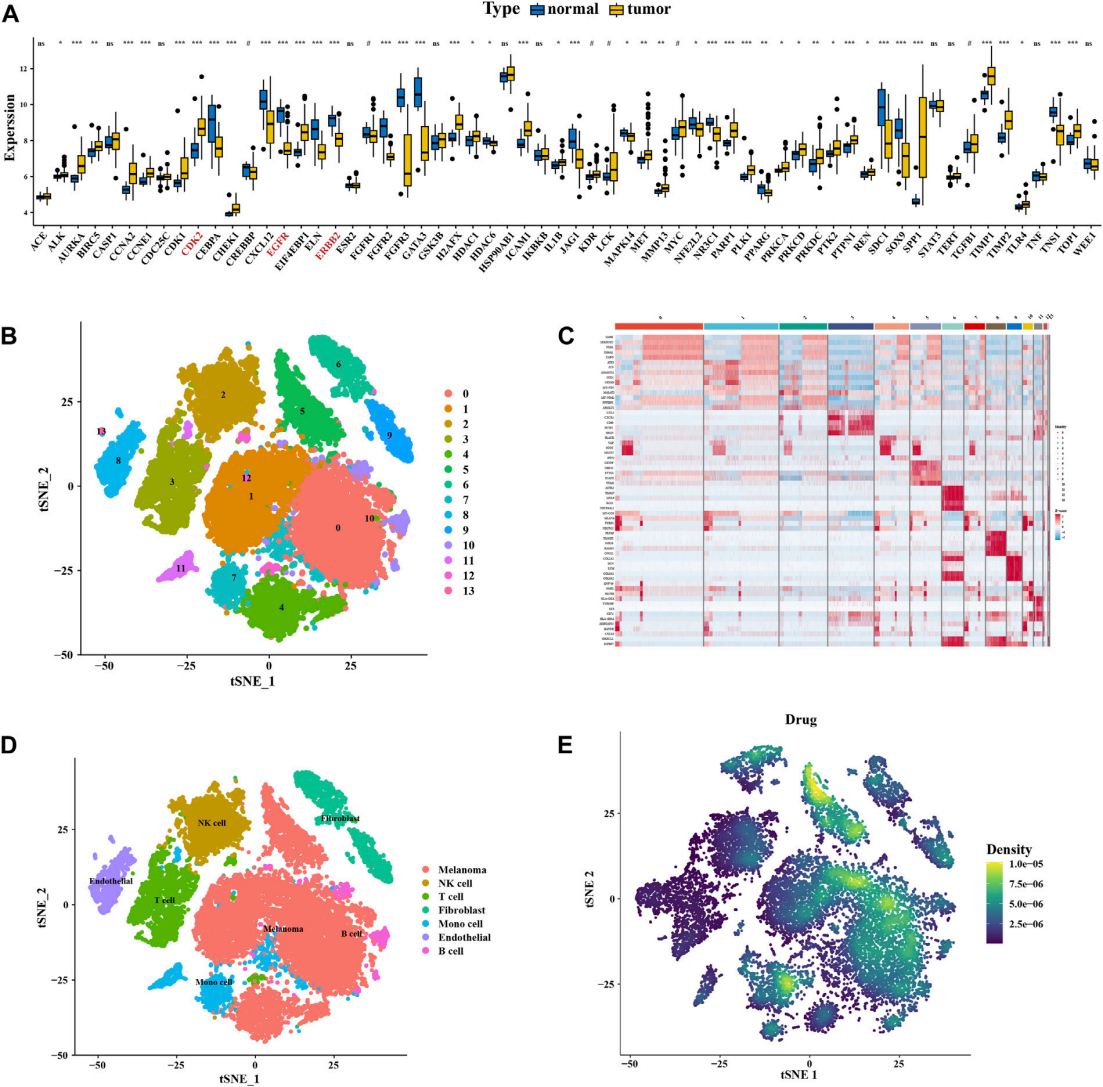
Expression and distribution of the key cluster. **(A)** Boxplot of differential expression of key genes in normal tissue and tumor tissue of data in GSE46517. **(B)** Unified manifold approximation and projection clustering into 13 clusters. **(C)** Heatmap of each gene table level. **(D)** Manual annotation of 13 clusters, finally identifying 7 clusters. **(E)** Melanoma drug pathways of action.

Differential expressions between the normal and disease samples of individual key targets were calculated and shown in box plots (Figure 5A). We then downloaded the single-cell data from the GEO database for our analysis. We used the following criteria to filter cells to guarantee their quality for our analysis: cells with > 5,000 and < 200 genes per cell and cells with a > 20% mitochondrial percentage or a > 20% ribosome percentage were filtered out (Supplementary Figure S3A). Then, the harmony package was used to remove the batch effect (Supplementary Figure S3B). The cluster tree was scaled with a resolution of 1.5 (Supplementary Figure S3C), and the principal component value was determined as 13. The results of T-distributed stochastic neighbor embedding (t-SNE) gathered 13 cell clusters (Figure 5B). The heatmap showed the gene type of each cluster (Figure 5C). Using the CellMarker database and referring to the work of Yang et al. (Zhang et al., 2022), we annotated these clusters into seven cells, including melanoma, NK cells, T cells, fibroblasts, mono cells, endothelial cells, and B cells (Figure 5D). The AUCell functional score analysis was used to show the distribution of drug targets, with cinobufagin acting mainly on melanoma cell clusters (Figure 5E).

### 3.6 Identification of crucial targets

**FIGURE 6:**
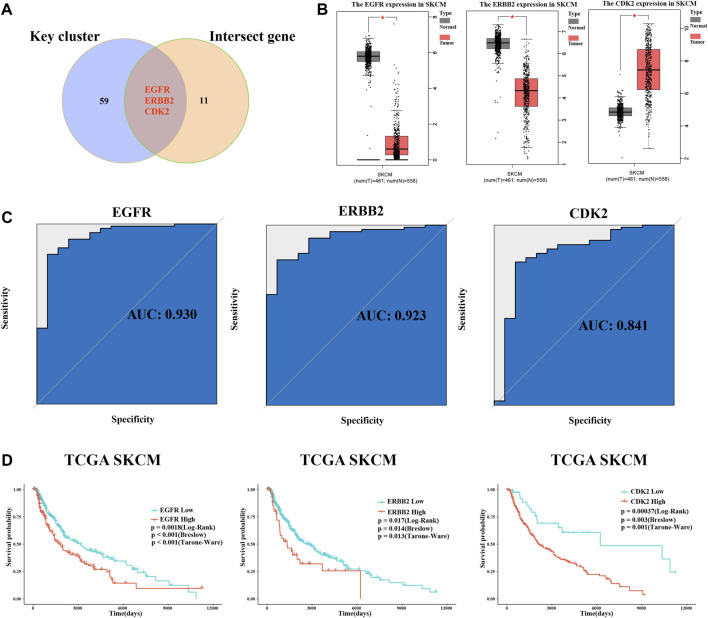
Identification of crucial targets, receiver operating characteristic curve (ROC) analysis, and survival analysis. **(A)** Intersection of key clusters and melanoma-related genes and drug targets, named crucial targets. **(B)** Differential expression of crucial targets in normal tissue and tumor tissue of data in TCGA database. **(C)** ROC curve of three crucial targets. **(D)** Survival curve of EGFR, ERBB2, and CDK2.

Through comparison with key targets and intersect genes from the intersection of drug targets and melanoma-related genes, we identified three crucial targets (Figure 6A). The ROC curves showed that all three had excellent robustness for melanoma (area under the ROC curve > 0.8) (Figure 6C). Moreover, melanoma patient survival data downloaded from the TCGA database (http://xena.ucsc.edu/) was used for survival analysis. Results showed that all three target genes had a significant impact on melanoma patient survival (Figure 6D). However, among these crucial targets, EGFR and ERBB2 were downregulated compared with the normal sample, and CDK2 was upregulated (Figure 5A). The expression data from TCGA melanoma show the same results (Figure 6B).

### 3.7 Molecular docking

**FIGURE 7:**
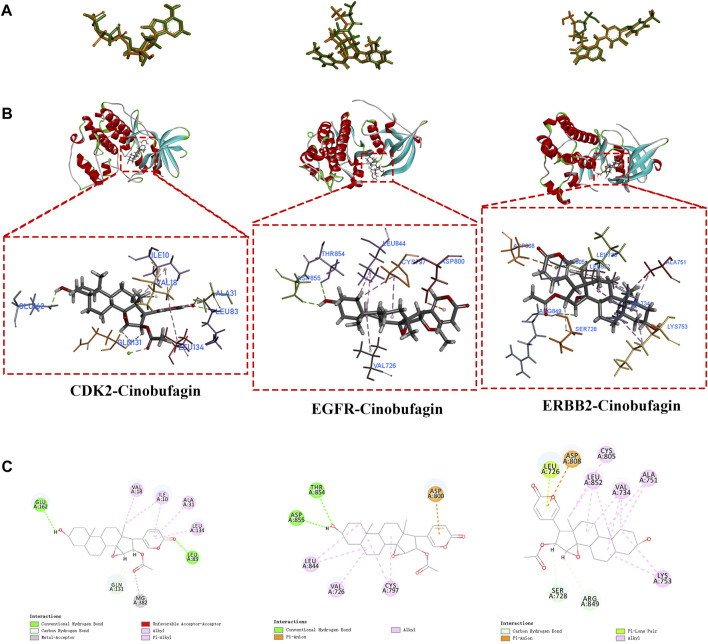
Molecular docking of crucial targets. **(A)** Re-docking of three targets in Discovery Studio 2019 software. **(B,C)** The CDOCKER results of cinobufagin with three crucial targets (CDK2, EGFR, ERBB2).

To validate the findings from network pharmacology, we selected crucial targets (CDK2, EGFR, and ERBB2) for molecular docking analysis to evaluate the screened targets. The structure of cinobufagin was identified by Chem Draw in 2022. After testing the feasibility of the docking method by redocking, the compound–target interactions, as well as their modes of binding, were visualized using Discovery Studio 2019 (Figures 7A–C). All these had high cdocker interaction energy, indicating that all three molecular docking targets combine very well with cinobufagin (Table1).

**TABLE 1:** Docking information on crucial targets.

Targets of cinobufagin	Re-docking (RMSD)	CDOCKER interaction energy (kcal/mol)	Binding energy (kcal/mol)
EGFR	0.7976	38.045	−43.5327
ERBB2	1.4621	25.9757	−21.7207
CDK2	1.1905	51.525	−62.8743

### 3.8 Molecular dynamics simulation

**FIGURE 8:**
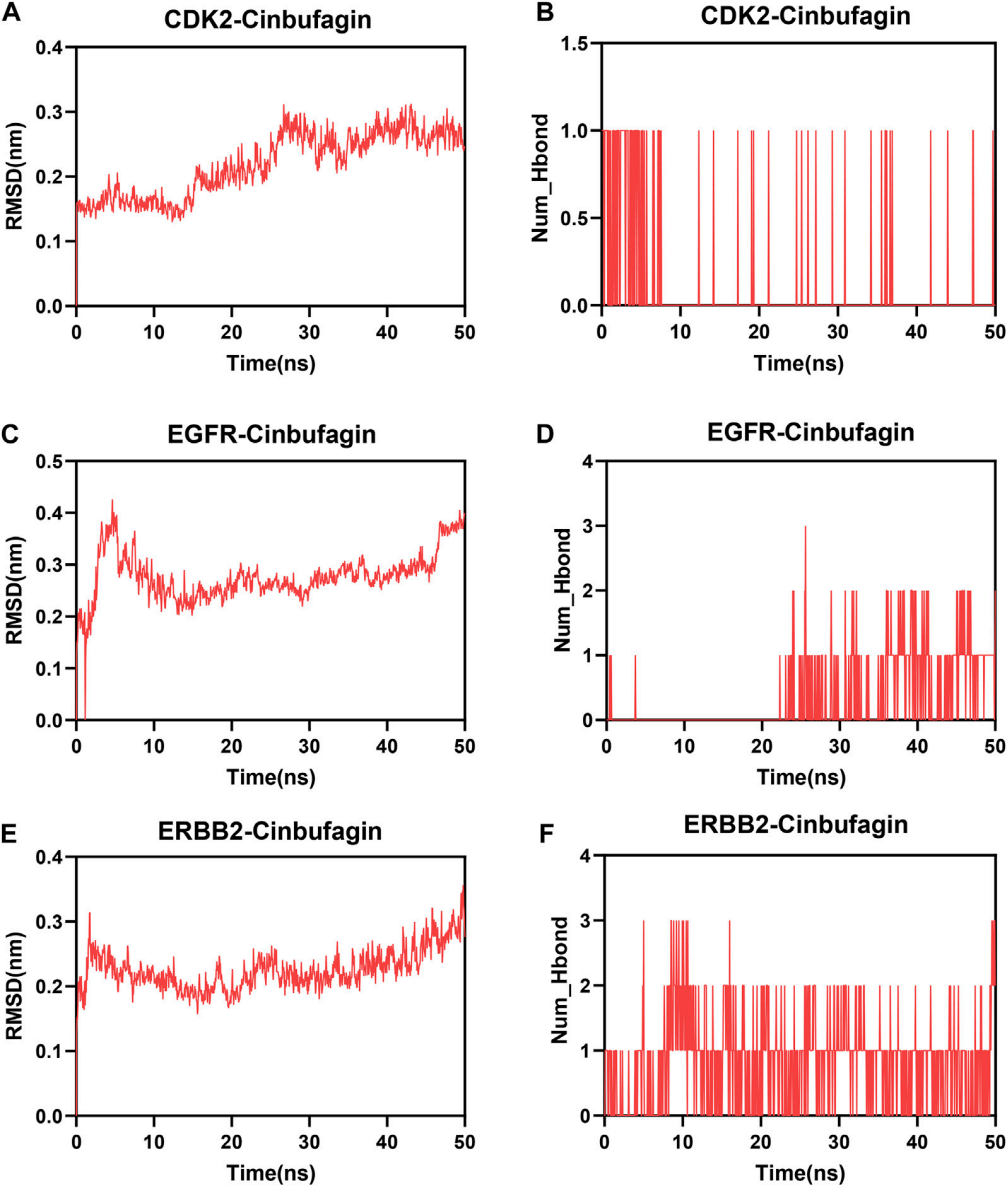
Molecular dynamics analysis. **(A,C,E)** Root mean square deviation (RMSD) of three systems (three crucial targets and cinobufagin). **(B,D,F)** Number of hydrogen bonds in three systems.

To further describe the binding patterns of protein–compound complexes, we performed molecular dynamics simulations of the above three molecular docking models. The RMSD curve can reflect the fluctuations of the system. As shown in Figure 8A, CDK2-cinobufagin was stable after 30 ns, and EGFR-cinobufagin and ERBB2-cinobufagin were stable after 10 ns (Figures 8C, E). The number of hydrogen bonds in the protein–cinobufagin complexes reflected their binding strengths (Figures 8B, D, F). Among them, ERBB2–cinobufagin had the highest hydrogen bond density and strength (Figure 8F). These data suggested that these three crucial targets interacted very well with cinobufagin in accordance with the molecular docking results.

## 4 Discussion

Malignant melanoma is considered the most aggressive skin cancer—more dangerous than other skin cancers. If not removed at an early stage, it can spread and metastasize rapidly. Thus, anticancer drug therapy is an important anti-melanoma therapy (Helmbach et al., 2001). However, standard chemotherapy does not produce satisfactory results due to chemotherapy resistance (Helmbach et al., 2001; Jilaveanu et al., 2009; Abildgaard and Guldberg, 2015; Kalal et al., 2017). The development of new, effective treatments for melanoma is thus vital.

Venenum bufonis is a traditional Chinese medicine that has been widely used in China (Zhang et al., 2012; Zhu et al., 2017). It has been reported that its extract can inhibit the growth of many tumor cells (Park et al., 2012). Cinobufagin is one of the active components of Venenum bufonis , which piqued our research interest. It is reported that cinobufagin can effectively inhibit the growth and development of lung cancer cells (Adjei et al., 2010), liver cancer cells (Cui et al., 2010), prostate cancer cells (Yu et al., 2008), and osteosarcoma cells (Dai et al., 2017) *in vitro*. Moreover, cinobufagin can also inhibit the proliferation of melanomas (Pan et al., 2019; Kim et al., 2020; Zhang et al., 2020). However, the underlying mechanism and potential targets of cinobufagin in melanomas are unclear.

In this study, we combined network pharmacology, bulk RNA sequencing data, and single-cell RNA sequencing data to finally identify the potential targets of cinobufagin in melanoma. First, we found and predicted the 413 potential targets of cinobufagin. It is interesting that the molecular function section comprised in GO was gathered in protein serine/threonine kinase activity, protein serine kinase activity, and protein tyrosine kinase activity. Then, we downloaded GEO data for DEGs and WGCNA analysis, finally identifying 329 disease-related genes. By inputting these disease-related and drug-related genes into the STRING online database, we constructed a PPI network. To further identify potential targets of cinobufagin in melanoma, we used the molecular complex detection (MCODE) algorithm to define a more important subset. Interestingly, the GO analysis of these key targets showed that the biological process category was enriched in the positive regulation of kinase activity, the regulation of protein serine/threonine kinase activity, and the molecular function category, showing that the key cluster was enriched in protein tyrosine kinase activity and protein serine kinase activity, which was in keeping with previous results. Furthermore, the KEGG analysis showed that these potential targets were mainly enriched in the cell cycle; it is reported that this pathway is one of the main effects of cinobufagin on cancer cells (Pan et al., 2019; Yang et al., 2021). We then downloaded single-cell data from the GEO database to verify the distribution of these key targets. The results show that 62 key targets were mainly gathered in melanoma cells. By intersecting the key cluster and the intersection of drug-related genes and melanoma-related genes, we finally identified three crucial targets, EGFR, ERBB2, and CDK2, which are all protein serine/threonine kinases and are involved in cell cycle regulation (Lo and Hung, 2006; Hirai et al., 2017; Kirova et al., 2022). It has been reported that cinobufagin can inhibit the EGFR-CDK2 signaling pathway in hepatocellular carcinoma, which is consistent with our predicted results (Yang et al., 2021).

The crucial targets we identified all have excellent robustness in melanoma. TCGA data indicate that these three crucial targets have a significant impact on melanoma patient survival by three test methods. It was confusing that EGFR and ERBB2 had low expression in tumor tissue compared to normal tissue whether in GEO or TCGA data, perhaps due to the disadvantage of bulk sequencing. Finally, we showed the molecular docking results of cinobufagin with these three crucial targets, and the molecular dynamics simulation was performed. These data suggest that docking of cinobufagin with three proteins is reasonable, indicating that these might be potential targets of cinobufagin in melanoma.

Network pharmacology is a practical strategy that uses computer technology to deepen our understanding of the modes of drug action across multiple scales of complexity (Hopkins, 2008). We combined network pharmacology with other sequencing data to identify key targets. Through single-cell sequencing analysis, we found that these key targets were mainly distributed in melanoma cells. We used molecular docking to show that the crucial targets were potential targets of cinobufagin in melanoma. Moreover, the results of our analysis have been partly verified in other tumors (Yang et al., 2021), indicating that this method has great value in drug target prediction.

It should be noted that this study had some limitations. First, sequencing data for our analysis were retrieved from the literature and databases; therefore, the reliability and accuracy of the predictions are dependent on data quality. The second is the absence of evidence to verify our predictions; clinical trials, animal experiments, and X-ray diffractometers are needed to confirm the findings. Third, experimental validation is necessary to further verify cinobufagin’s ability to bind and inhibit crucial targets, such as affinity assays (surface plasmon resonance (SPR) or isothermal titration calorimetry (ITC)) or direct mutation studies. Our conclusions remain preliminary as long as computational predictions are not supported by experimental validation.

## Data Availability

The datasets presented in this study can be found in online repositories. The names of the repository/repositories and accession number(s) can be found in the article/Supplementary Material.
