# Supplementary material for: Mechanisms underlying the therapeutic effects of cinobufagin in treating melanoma based on network pharmacology, single-cell RNA sequencing data, molecular docking, and molecular dynamics simulation
Source: Front Pharmacol. 2024 Jan 29;14:1315965. doi: 10.3389/fphar.2023.1315965 (PMC10859445; doi:10.3389/fphar.2023.1315965)
Supplement: Supplementary file 1 [file DataSheet1.PDF]

Supplementary Material

Mechanisms underlying the therapeutic effects of Cinobufagin in treating melanoma based on network pharmacology and single-cell RNA sequencing data and molecular docking

Jiansheng Yang *, Chunchao Cheng, Zhuolin Wu

*** Correspondence: Jiansheng Yang**: 16622593047@163.com

Supplementary Figures

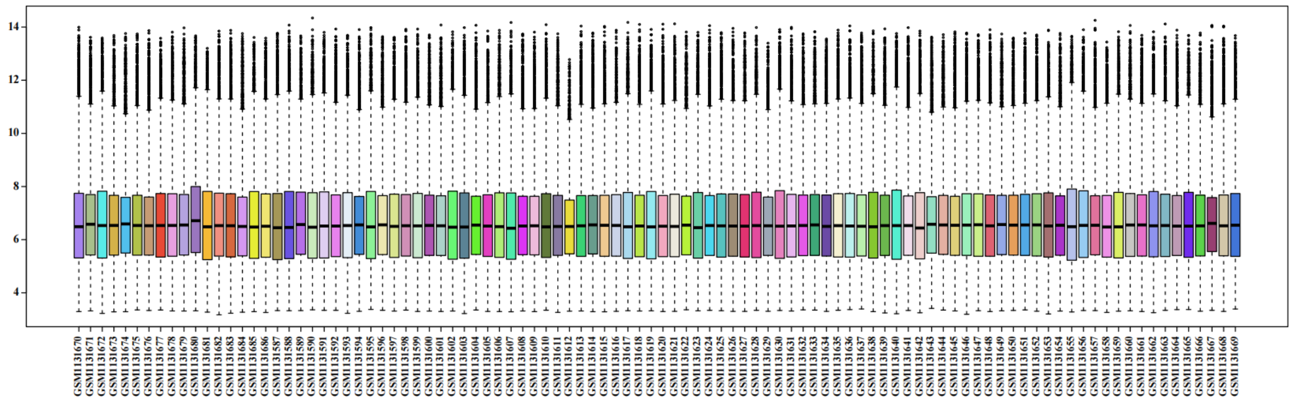

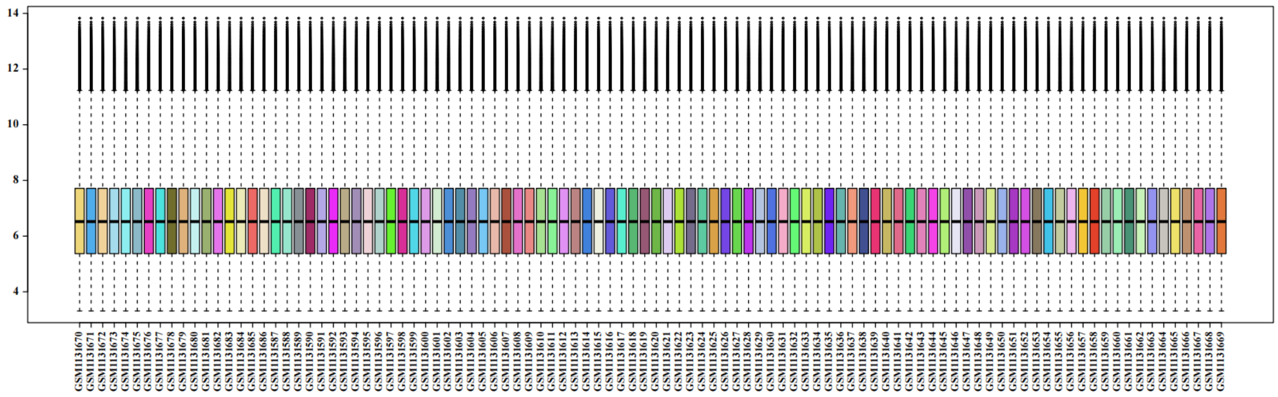

Supplementary Figure 1. The data normalization of GSE46517

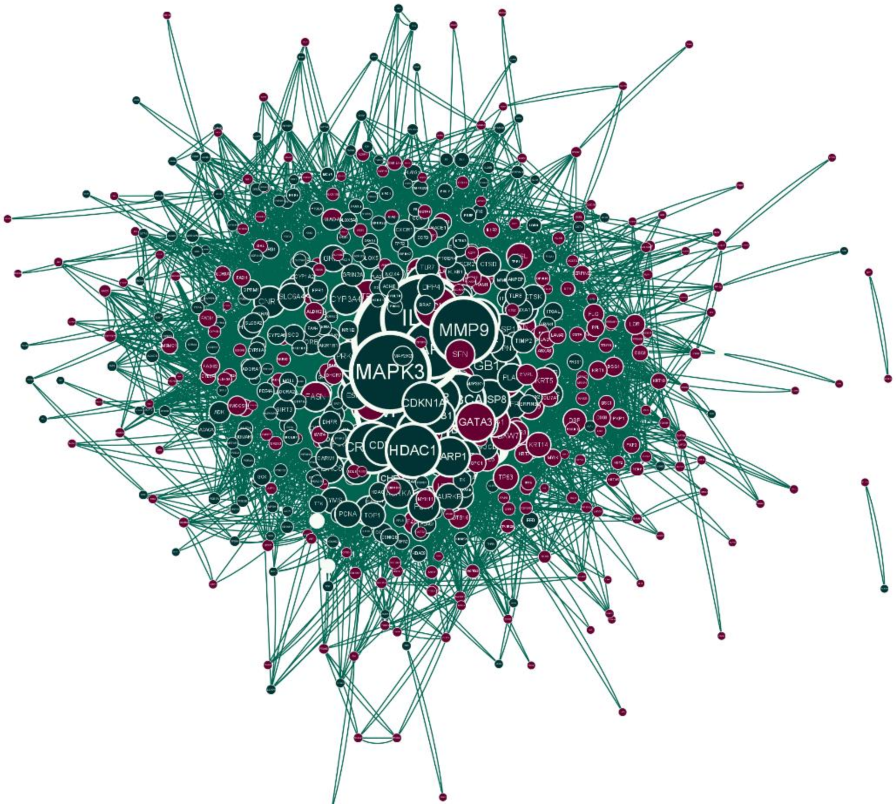

Supplementary Figure 2. The protein-protein interaction network of all drug targets and melanoma-related genes.

A

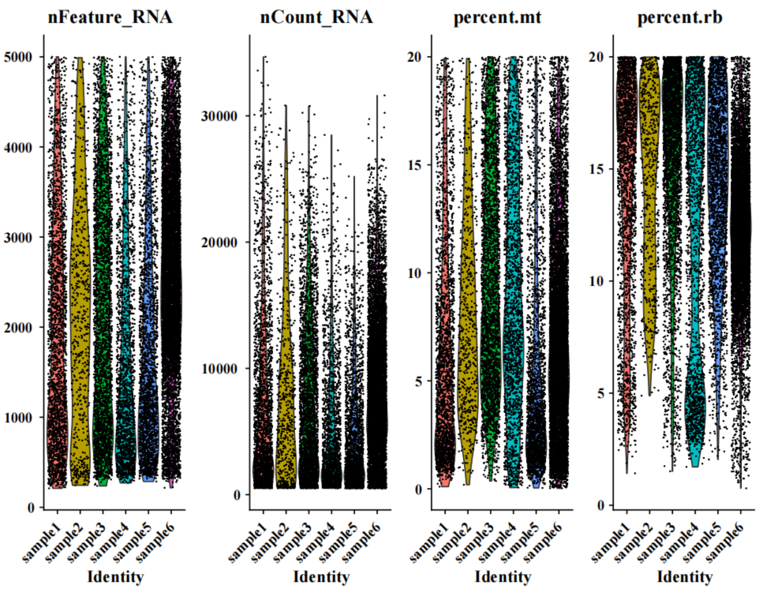
nFeature_RNA
nCount_RNA
percent.mt
percent.rb
Identity
Identity
Identity
Identity

B

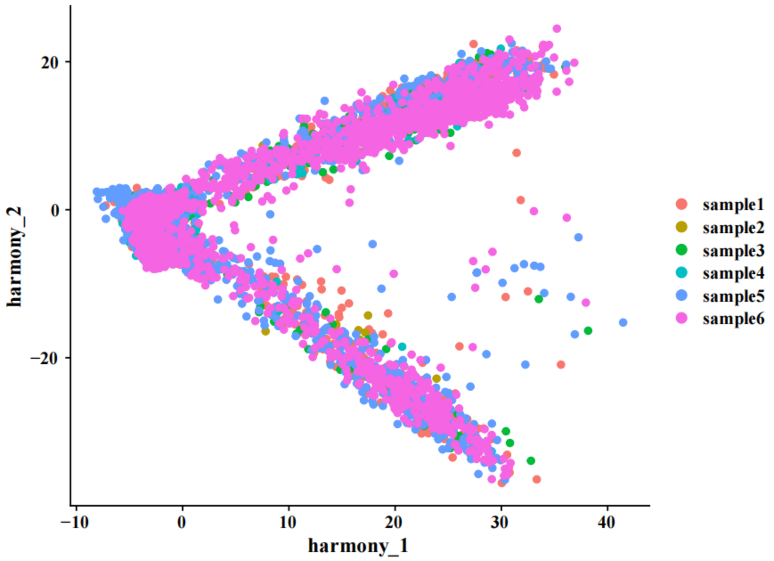
harmony_2
harmony_1
sample1
sample2
sample3
sample4
sample5
sample6

C

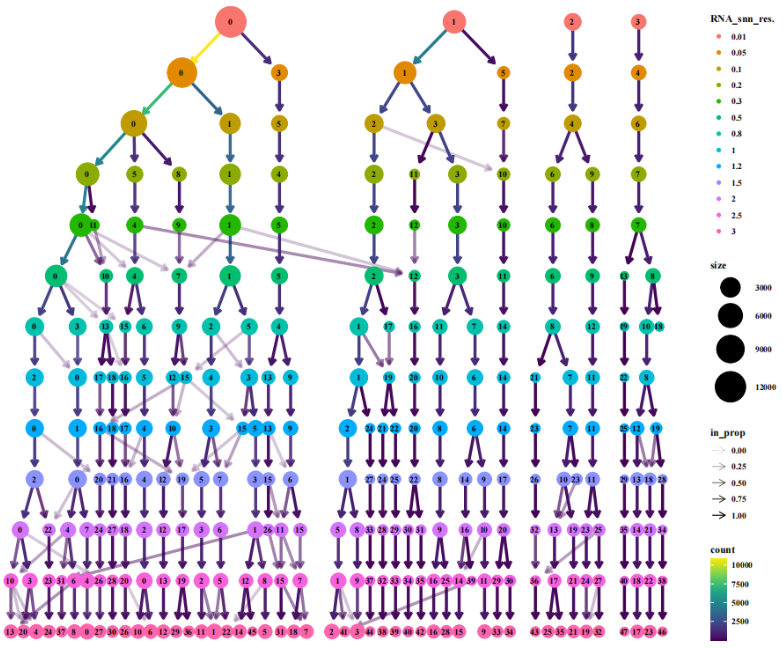

Supplementary Figure 3. The data normalization of GSE215120. (A) The filtered principal of min.cells = 3, min.features = 200, af$nFeature_RNA >= 200 & af$nFeature_RNA <= 5000, af$percent.mt <= 20, af$percent.rb<= 20. (B) After remove batch effect. (C) The cluster tree was scaled to a resolution of 1.5.

Supplementary Table 1. The differential expression genes and fold change.

DEGs	logFC
HLF	-1.02595
CDHR1	-2.52834
FGFR2	-1.50453
EPB41L4B	-1.35944
PHYHIP	-1.13138
CHP2	-1.95825
PLLP	-1.23426
WIF1	-1.17572
MAOA	-1.28848
PDZD2	-1.4889
EXPH5	-2.27636
RNASE4	-1.08267

TGFBR3	-1.56269
FBXW7	-1.80013
CLEC3B	-2.79493
SCEL	-2.24623
PARD3	-1.2988
CCL27	-2.61871
PRAME	3.459098
ZBTB16	-1.75581
NFIB	-1.75775
ARPC1B	1.315706
NDRG2	-1.48009
ALDH3A2	-1.40483
ASS1	-2.17265
MYH11	-2.08251
SCGB1D2	-2.9324
AFF1	-1.01416
EGFR	-1.7384
SCGB2A2	-3.86291
POU2F3	-1.41183
TNXA	-2.05079
CNN1	-1.87261
EFS	-1.06922
RAPGEFL1	-1.8427
SCNN1B	-1.07199
DLG5	-1.58758
ADH1B	-1.64186
WNT4	-1.59317
OSR2	-1.62296
PLAGL1	-1.34981
KRT19	-1.77383
MYLK	-1.8224
LMOD1	-1.31529
C1orf116	-1.64253
ANK3	-1.30867
SOWAHC	-1.14229
GATA3	-2.69214
TACC2	-1.45091
COL17A1	-2.39198
FAM57A	-1.18979
RORA	-2.41695
CXADR	-2.74132
C14orf132	-1.06281
FGFR3	-3.45248

COBL	-1.25754
KRT15	-4.4544
PLEKHA1	-1.09086
EPHB6	-1.86469
IL11RA	-1.2899
CFD	-2.71189
CTSB	1.23314
LRIG1	-1.17418
FAT2	-1.2265
NTRK2	-1.20165
KRT7	-1.10693
FBLN1	-1.5627
PAMR1	-1.04388
PPP1R13L	-1.46028
RXRA	-1.32186
ID4	-1.60925
TMEM8A	1.218649
DUSP4	2.012078
KLF5	-2.8671
ZNF395	-1.1954
SDC4	-1.46403
AACS	-1.52394
DES	-1.506
GAS6	-1.08495
FHL1	-1.86214
ACSL1	-1.55232
ACTG2	-1.73673
NUAK1	-1.17014
FBXO16	-1.17746
JAG2	-1.18398
PIP	-1.99589
OLFML2A	-1.03511
MARCKSL1	1.294418
CD207	-1.93124
SLC20A1	2.01456
PALMD	-1.46981
ANO1	-1.10147
LRRC59	1.056668
DNM1	-1.18017
INSR	-1.07419
BBOX1	-1.78916
NOTCH3	-1.1004

ELN	-1.1842
EVPL	-1.32238
CYP4B1	-1.03396
PAK6	-1.06059
STARD5	-1.05922
FKBP11	1.667851
DAAM1	-1.33782
ERBB2	-1.05479
FERMT1	-1.26146
DEGS1	-1.22222
ADIRF	-1.8745
ZSCAN18	-1.33817
COTL1	1.483015
EN1	-1.03487
ECHDC2	-1.07032
ACOT7	1.195722
PTPRF	-2.02607
IRF6	-1.25969
FADS2	-2.23207
FCGBP	-1.8038
DHCR24	-1.80551
C1orf106	-1.56137
CA12	-2.25162
CHCHD3	1.064421
UPP1	1.44551
UBE2S	1.346821
CTNNBIP1	-1.01608
ACSBG1	-1.87929
VAV3	-1.18829
CFH	-1.80849
IL37	-1.3301
SH3GLB2	-1.04443
DSC3	-2.88756
FCER1A	-1.9851
TPM2	-1.47604
ALOX15B	-1.96998
PHACTR1	2.112782
TPPP3	-1.46569
POU3F1	-1.10674
C1orf68	-1.38647
KRT31	-1.35658
IFI16	1.076743
PLOD3	1.266152

NET1	-1.28695
GJB5	-1.05556
TP63	-2.17096
HMGCS1	-1.13645
AZGP1	-1.70315
BCL11B	-1.16949
MYL9	-1.60797
AHNAK	-1.46315
ITM2A	-1.16737
WARS	1.880842
SNX10	1.917139
CTSZ	1.34236
GATM	-1.36206
SMAD1	-1.30046
C9orf3	-1.23761
SPINT1	-1.32946
HSD11B1	-1.12913
TUFT1	-1.46874
CTSL	1.183436
EMP2	-1.45215
ETS2	-1.09989
AKR1C1	-1.1835
AKR1C3	-1.11918
AHNAK2	-2.80954
ABLIM1	-1.66673
C1QB	2.15507
ANXA8	-2.44201
KLK11	-1.97244
BCL6	-1.00635
CEBPA	-1.34736
GSTA4	-1.19394
SPP1	3.506211
PPL	-2.56448
CHRDL1	-1.08999
KRT23	-1.94595
TYMS	1.503224
DSC1	-2.68914
PRSS8	-1.14122
H2AFZ	1.052261
AKR1C2	-1.14901
TMEM47	-1.84101
EIF4EBP1	1.121901

DEFB1	-1.46746
ALDH3A1	-1.2927
UBE2C	1.004464
KLF4	-1.96842
LEF1	1.456734
GDF15	3.092942
BMP2	-1.12891
CRIP1	-1.90519
CHST11	1.134659
PTTG1	1.068603
PTPRK	-1.10338
TRIB2	1.446932
SORBS2	-1.00616
TIMP1	1.074532
GAS1	-1.04258
C12orf5	1.077996
RGS1	2.409615
KPNA2	1.248529
PKP1	-2.82768
TM7SF2	-1.11575
COX7A1	-1.03272
SERPINE2	2.035789
SH3YL1	-1.2181
MMP7	-1.08298
IMPA2	-1.87526
PDGFRL	-1.13953
AUTS2	-1.48771
SLC25A32	1.35859
SH2B3	1.16128
ITGB4	-1.74364
IFI30	1.237772
SPINT2	-1.58749
FBLN2	-1.24747
HOPX	-2.47078
LGALS3BP	1.282781
PTGS1	-1.16982
ZNF750	-1.52288
LGALS7	-3.47814
S100A1	1.962547
MSMO1	-1.64058
TAGLN	-1.54983
DKK3	-1.61273
MFAP4	-1.16985

DPT	-1.22752
DHCR7	-1.25271
CCNB1	1.30938
LPXN	1.213745
KRT2	-2.7809
DBF4	1.002879
MAGEA3	2.533926
GJB3	-1.06392
CKMT1A	-1.61708
GAL	-1.81011
HLA-DQB2	-1.80247
GPR87	-1.24506
FCGR2A	1.117299
GPC1	-1.188
TPX2	1.018155
CYB5A	-1.0094
HEBP2	-1.18231
TRIM29	-2.76317
FXYD1	-1.04263
TNFRSF21	1.022672
METTL7A	-1.1771
AQP1	-1.50448
CCL14	-1.21916
TACSTD2	-3.01219
TFAP2B	-1.87976
FABP7	-1.75287
DUOX1	-1.60021
HEY1	1.285593
JAG1	-1.01902
CALU	1.030784
CYP26B1	-1.33071
ALDH2	-1.61979
DSG1	-3.00423
IL1R2	-1.16927
STXBP1	1.115537
MAGEA6	2.190286
PLAT	1.694088
LAMA3	-1.02933
KIAA0101	1.410743
EFEMP1	-1.25973
SDC1	-1.59941
FA2H	-1.28545

CDK2	1.146116
TNC	1.346148
SERPINB5	-2.58365
NUSAP1	1.023261
TIAM1	-1.15495
MMP9	1.560545
RRM2	1.457139
CKS2	1.532659
NMU	-1.08448
EDNRB	1.719036
AQP3	-1.57844
TNS1	-1.06539
LY6D	-2.33389
HCAR3	-1.09157
AIM1	-1.34379
DSP	-3.11747
ATP6V1C1	1.098545
S100A14	-1.72579
HBB	-1.09948
PKP3	-1.50468
PHLDA2	1.436274
MYOF	-1.09497
ACKR1	-1.22745
MAP1B	-1.07006
ZWINT	1.041622
IL1RN	-1.14564
LOC101928198	-1.12047
STAT1	1.198775
SLC45A2	1.078811
TGFBI	-1.13342
CITED1	1.02841
ID1	-1.12126
RAB25	-1.96487
LOC100506558	-1.1322
CXCL14	-2.51142
MIA	1.381925
FADS1	-1.13671
KRT1	-3.31529
DUSP6	1.068218
WIPI1	1.183065
EFNB2	-1.03479
SLC7A5	1.204828
MXRA5	-1.08057

PERP	-2.25403
C1QA	1.344252
HBA1	-1.49156
TPSAB1	-1.40501
KRT5	-3.21585
LAD1	-1.16887
FASN	-1.0305
CXCL9	2.03679
DCN	-1.26423
CXCL12	-1.25928
RGS20	1.354479
TPSB2	-1.51454
DTL	1.049487
ALDH3B2	-1.54663
DSG3	-1.34366
PALLD	-1.00277
MAGEA12	1.606237
CA6	-1.33597
IGFBP5	-1.1338
IFI6	1.20064
CD9	-1.02586
PLEKHB1	1.17685
TOP2A	1.106505
CST6	-2.19578
PTN	-1.07232
NRIP3	1.01287
CLCA2	-1.56578
FAIM3	1.051837
GJA1	-1.86675
KRT14	-3.99475
KRT10	-1.49906
SFN	-2.14076
MTHFD2	1.081065
PPAP2B	-1.11205
LY6G6C	-1.19282
CCL5	1.295281
CLIC3	-1.4696
CXCR4	1.02269
S100B	1.605168
MAD1L1	1.070405
AP1S2	1.05291
CA2	-1.08853

CCL18	1.184724
BST2	1.115476
SOX9	-1.25403
TCN1	1.013054
FRZB	-1.18641
TMEM45A	-1.81458
SELL	1.206192
SERPINA1	1.049584
LCE2B	-1.5552
EPHX3	-1.08335
TUBA4A	-1.32287
ABCA8	-1.01227
ADAMDEC1	1.195464
LOR	-2.3688
KLK5	-1.25263
PBK	1.00819
LYPD3	-1.53156
SPINK5	-1.49404
TNFSF10	-1.00598
SPARCL1	-1.04099
CXCL10	1.284372
CALML3	-1.65297
GPX3	-1.12489
BAMBI	1.115349
CLU	-1.23751
PLA1A	1.276254
IGHG1	1.882109
GPR143	1.06745
FLG	-1.91051
CSTA	-1.84723
ISG20	1.069871
CXCL13	1.284853
CDH19	1.105683
BAALC	1.019003
GBP1	1.103824
CDH1	-1.26522
CRCT1	-1.06015
CALML5	-1.45933
